# Longitudinal Associations Between Breakfast Consumption, Sleep Duration and Depressive Symptoms in Adolescents

**DOI:** 10.3390/nu18142252

**Published:** 2026-07-10

**Authors:** Xiaoyan Yu, Yuxun Peng, Sihan Jing, Jingfen Zhu

**Affiliations:** 1 Department of Nutrition, Shanghai Chest Hospital, Shanghai Jiao Tong University School of Medicine, Shanghai 200030, China; 2 School of Public Health, Shanghai Jiao Tong University, Shanghai 200025, China

**Keywords:** depressive symptoms, breakfast consumption, sleep duration, adolescents

## Abstract

**Objectives:** This study aimed to investigate the longitudinal associations between breakfast consumption, sleep duration and depressive symptoms among adolescents. **Methods:** The baseline survey (T1) was conducted from November to December 2019 using a multi-stage stratified cluster sampling method among secondary school students in Shanghai, China. The follow-up survey was conducted about one and a half years later (T2). A total of 2502 adolescents were included in the final analysis. Depressive symptoms were evaluated using the Chinese version of the Patient Health Questionnaire-2 (PHQ-2-C). Breakfast consumption frequency and sleep duration in the past week were self-reported. A cross-lagged model was constructed to examine the longitudinal associations between breakfast consumption, sleep duration and depressive symptoms. **Results:** The results showed that the prevalence of depressive symptoms and insufficient sleep increased from 15.31% and 89.53% at T1 to 18.47% and 92.89% at T2, respectively. The rate of daily breakfast consumption decreased from 81.10% to 76.06%. The cross-lagged model showed that daily breakfast consumption could significantly predict depressive symptoms (*β* = −0.109, SE = 0.030, *p* < 0.001) and sleep duration (*β* = 0.070, SE = 0.028, *p* = 0.013). Sleep duration could predict depressive symptoms (*β* = −0.076, SE = 0.022, *p* < 0.001) and vice versa (*β* = −0.039, SE = 0.018, *p* = 0.028). **Conclusions:** The rate of daily breakfast consumption among adolescents decreased, alongside the prevalence of depressive symptoms and insufficient sleep increased. Daily breakfast consumption predicted depressive symptoms and sleep duration, whereas depressive symptoms and sleep duration may have a bidirectional association.

## 1. Introduction

Adolescence, defined by the World Health Organization as ages 10–19, is a unique stage of human development and a crucial period for laying the foundation for physical and mental health, as well as for establishing behavioral patterns such as diet, physical activity, and substance use [1]. In recent years, adolescent mental health problems have become a significant concern in global public health. Globally, approximately 14% of adolescents aged 10–19 years experience mental health conditions, yet these remain largely unrecognized and undertreated [2]. Depression is one common mental health problem. The incidence of depressive symptoms among adolescents aged 10–19 years is about 34% worldwide, and shows an upward trend [3]. The prevalence of depression symptoms among Chinese children and adolescents is about 13.06–15.27% [4,5]. Depression among adolescents can lead to severe consequences, such as compromised social functioning, insomnia, substance use, and obesity, and is significantly associated with an increased risk of suicide [6]. Adolescent depression may be influenced by multiple factors, including genetic factors, negative life events, family-related factors (e.g., parent–child communication, parental over-involvement, family functioning and family cohesion) and school-related factors (e.g., academic performance, peer support, peer rejection, and peer relationships) [6,7].

In addition, lifestyle factors such as skipping breakfast and short sleep duration are all associated with the risk of depressive symptoms, and lifestyle risk factors seem to have an additive effect on mental health [8]. Regular breakfast intake is beneficial to the physical and mental development of adolescents, such as improved nutritional status, cognitive function and mental health. Moreover, breakfast habits established in childhood and adolescence may persist into adulthood [9]. However, skipping breakfast has become one of the common unhealthy eating behaviors among adolescents. A systematic review showed that 10–30% of children and adolescents skipped breakfast [10]. A national survey in China indicated that nearly 15% of adolescents aged 12 to 17 do not eat breakfast every day [11]. Meanwhile, the sleep problems of adolescents are also a matter of concern. Numerous adolescents currently fail to meet the recommended sleep duration. Research indicated that 77.7% of U.S. adolescents do not obtain the recommended eight hours of sleep on weekdays [12]. A previous report showed that 26% of adolescents in Canada do not meet the recommended sleep duration [13]. The situation seems also concerning in some Asian countries. The average weekday sleep duration of Korean adolescents was even less than 5 h per day [14]. In China, research has indicated that 24.75% of adolescents aged 14–17 fall within the 8–10 h sleep duration range [15]. Another report showed that the average sleep duration of Chinese adolescents was 7.8 h on workdays in 2020, which was 0.3 h lower than that in 2009 [16].

Several cross-sectional studies have indicated a significant negative association between breakfast consumption and depressive symptoms in adolescents [4,17,18]. The mechanism by which breakfast affects depressive symptoms is unclear. A plausible explanation is that breakfast commonly includes foods such as grains, eggs, and dairy products, which are rich in nutrients like tryptophan, magnesium, and calcium that play crucial roles in mood regulation and neural function [9]. Meanwhile, skipping breakfast may impair cognitive function, lead to a decline in academic performance [19], or cause overweight and obesity [20], which in turn may trigger more mental health problems. Unfortunately, the results of longitudinal studies in adolescents have been inconsistent [21,22,23]. On the other hand, depressive symptoms might also have an impact on breakfast. Depressive symptoms may lead to alterations in individual appetite, and appetite changes are also common in adolescents with severe depression [24]. However, the longitudinal evidence regarding the impact of depressive symptoms on breakfast among adolescents remains limited. Therefore, additional longitudinal studies are necessary to further elucidate the mutual relationship between breakfast and depressive symptoms.

There may be a bidirectional association between sleep problems and mental health issues. Sleep duration is an important influencing factor for depressive symptoms, and insufficient sleep increases the risk of depressive symptoms in adolescents [4]. Sleep deprivation may contribute to emotional disorders by reducing pleasurable feelings, impairing the function of brain regions involved in emotion regulation (e.g., weakened prefrontal-limbic connectivity), disrupting the processing of emotional memories during rapid eye movement sleep, or undermining the cognitive functions that support emotion monitoring and regulation [25]. Additionally, depressive symptoms may also lead to sleep problems. A case–control study indicated that the sleep quality of adolescents with depression was inferior to that of healthy controls [26]. Individuals with depression often use rumination as a negative coping style [27], which might lead to more sleep problems [28]. However, the findings of longitudinal studies are inconsistent. Some studies have observed a bidirectional association between sleep and depression [29,30]. However, another study has shown that the longitudinal association between sleep duration, weekend screen time and internalizing symptoms is unidirectional (behavior precedes internalizing symptoms) and only exists among girls [31]. On the contrary, Lovato et al. found that depressive mood might be the antecedent of poor sleep, but the reverse was not predictive [32].

Moreover, sleep duration exhibits significant associations with multiple lifestyle factors, including breakfast consumption among adolescents [33]. Cross-sectional studies have demonstrated that there is a negative association between sleep duration and breakfast consumption among adolescents [34,35]. Insufficient sleep may be an independent factor for skipping breakfast in adolescents [36]. A worse sleep health predicts less frequent breakfast consumption among adolescents in a micro-longitudinal analysis [37]. Conversely, breakfast consumption may also have a positive effect on sleep duration [38].

Notably, breakfast, sleep, and depression are all associated with the circadian rhythm. The circadian rhythm system regulates the temporal patterns of nearly all human behaviors and physiological activities. Both dietary intake and sleep affect circadian rhythm adjustment, and breakfast, being the first meal after the longest fasting period of the day, may be particularly important in affecting circadian rhythms [39]. Furthermore, circadian rhythm disruptions and mood disorders exhibit a bidirectional relationship, suggesting that circadian irregularities may serve as both precursors and risk factors for mood episodes [40]. Although existing studies have demonstrated close relationships among breakfast, sleep, and depressive symptoms in adolescents, most studies are cross-sectional. Longitudinal studies remain limited. Therefore, this study aimed to examine the longitudinal associations between breakfast consumption, sleep duration and depressive symptoms in adolescents.

## 2. Materials and Methods

### 2.1. Study Design and Participants

This study employed a two-wave longitudinal survey among secondary school students in Shanghai, China. The baseline survey (T1) was conducted from November to December 2019. Schools were first stratified by region (urban and suburban) and then by school type (junior and senior high school), resulting in a total of 14 participating schools (10 junior high schools and 4 senior high schools). Participants were junior high school students in grades 6 and 7 (aged 11–13 years) and senior high school students in grade 10 (aged 15–16 years), totaling 3064 students at baseline. The follow-up survey (T2) was conducted from April to May 2021, following up on the baseline sample. A total of 2896 students were surveyed in T2. As the survey was conducted anonymously, the two-wave data were matched using key identifiers such as school, class, sex, and birthday, yielding 2643 matched students. After excluding 141 questionnaires with missing key information, invalid questionnaires or responses shorter than 240 s, a total of 2502 students were ultimately included.

### 2.2. Measures

#### 2.2.1. Depressive Symptoms

The Chinese version of the Patient Health Questionnaire-2 (PHQ-2-C) [41] was used to screen and evaluate depressive symptoms. The questionnaire used a 4-point Likert scale with 2 items ranging from 0 (not at all) to 3 (almost every day). Total scores range from 0 to 6, with a score ≥3 indicating the presence of a depressive disorder. Depressive symptoms were measured at both T1 and T2. The reliability and validity of the PHQ-2-C have been well established [41]. In this study, Cronbach’s α for the scale was 0.855 at T1 and 0.849 at T2.

#### 2.2.2. Breakfast Consumption

Breakfast consumption was assessed by the following question: “How many days did you eat breakfast in the past 7 days?”. Response options included “0 days,” “1 day,” “2 days,” “3 days,” “4 days,” “5 days,” “6 days,” and “7 days.” Participants who responded ‘7 days’ were placed into the daily breakfast consumption category. This question was asked at both T1 and T2.

#### 2.2.3. Sleep Duration

Sleep duration was assessed using a semi-quantitative method in which participants were asked the following questions: “How many hours do you usually sleep on weekdays (school days) last week?” and “How many hours do you usually sleep on the weekend last week?” The interval median conversion method was used to convert the median of each interval into quantitative values, which were 6.5 h, 7.5 h, 8.5 h, and 9.5 h, respectively. Average daily sleep duration was calculated as follows: (weekday sleep duration × 5 + weekend sleep duration × 2)/7. In accordance with the sleep guidelines issued by the Ministry of Education of the People’s Republic of China [42], junior high school students who sleep less than 9 h and senior high school students who sleep less than 8 h per day were defined as having insufficient sleep. This question was asked at both T1 and T2.

### 2.3. Covariates

The following information was also included in the study: sex (girl/boy), school type (junior/senior high school), monthly pocket money (below ¥200, ¥200–599, ¥600 or above), academic performance (top quarter, middle, and bottom quarter), parental education level (junior high school or below, senior high school or equivalent, junior college or equivalent, bachelor’s degree or above). Body mass index (BMI) was calculated by dividing weight in kilograms by height in meters squared and then classified into three groups based on Chinese age- and sex-specific screening standards for children and adolescents [43,44]: “Underweight,” “Normal,” and “Overweight or Obese”. Screen time (TV/videos, internet and mobile phone use) was dichotomized as ≥2 h/day and <2 h/day based on any single activity reaching 2 h per day. Physical activity was derived from the question: “During the past week, on how many days were you physically active for a total of at least 60 min per day (e.g., walking, jogging, playing balls, swimming, biking, or doing housework)?” and divided into 7 days/week and <7 days/week. These variables were asked at T1.

### 2.4. Statistical Analysis

The statistical analyses were performed using SPSS (version 26, IBM Corp., Armonk, NY, USA) and Mplus (version 8.3, Muthén & Muthén, Los Angeles, CA, USA). Descriptive statistics and Spearman correlation analysis were conducted in SPSS. Categorical variables were presented as frequencies (*n*) and percentages (%). Continuous variables were expressed as mean and standard deviation (M ± SD). McNemar’s tests were used to analyze differences between two waves of data, stratified by sex and school type. Spearman correlation analysis was performed to explore the relationships among depressive symptoms, breakfast consumption, and sleep duration. A cross-lagged model was conducted by Mplus. As breakfast consumption was a binary variable, parameter estimation was conducted through the weighted least squares mean and variance adjusted (WLSMV) method in the cross-lagged model. The cross-lagged model was saturated (df = 0). Therefore, model indices were no longer reported. Only path coefficients were of concern. In Spearman correlation analysis and the cross-lagged model, depressive symptoms and sleep duration were analyzed as continuous variables. Depressive symptoms and sleep duration were represented by the total score and quantified values (hours), respectively. All statistical tests were two-tailed, with *p* < 0.05 considered statistically significant.

## 3. Results

### 3.1. Description of Demographic Variables

The mean age of 2502 students at baseline (T1) was 12.97 ± 1.69 years. Among them, 1260 (50.36%) were girls and 1242 (49.64%) were boys. The proportions of junior high school and senior high school students were 70.66% and 29.34%, respectively. The distribution of the remaining variables is shown in Table 1.

### 3.2. Changes in Daily Breakfast Consumption, Sleep Duration and Depressive Symptoms

Table 2 shows that from T1 to T2, the rate of daily breakfast consumption among adolescents decreased from 81.10% to 76.06% (*p* < 0.001), while the prevalence of depressive symptoms and insufficient sleep increased from 15.31% and 89.53% at T1 to 18.47% and 92.89% at T2, respectively (*p* = 0.001 and *p* < 0.001).

After grouping by sex, a decrease in the rate of daily breakfast consumption and an increase in the prevalence of insufficient sleep were observed among both boys and girls (both *p* < 0.01), whereas a significant increase in the prevalence of depressive symptoms was observed only in boys, rising from 13.45% at T1 to 17.07% at T2 (*p* = 0.005).

Additionally, junior high school students reported a decrease in the rate of daily breakfast consumption from 83.43% at T1 to 77.83% at T2, while the prevalence of depressive symptoms and insufficient sleep increased from 13.74% and 89.71% at T1 to 17.31% and 94.17% at T2, respectively (*p* = 0.002 and *p* < 0.001). However, changes among senior high school students were not significant (*p* > 0.05).

### 3.3. Correlation Analysis

The results indicated that daily breakfast consumption, sleep duration and depressive symptoms exhibited significant correlations between T1 and T2 (*p* < 0.001). Depressive symptoms T1 was negatively correlated with daily breakfast consumption T1, daily breakfast consumption T2, sleep duration T1 and sleep duration T2 *(r* = −0.195, −0.130, −0.264 and −0.202, *p* < 0.001). Depressive symptoms T2 was also negatively correlated with daily breakfast consumption T1, daily breakfast consumption T2, sleep duration T1 and sleep duration T2 (*r* = −0.156, −0.220, −0.190 and −0.256, *p* < 0.001). Daily breakfast consumption T1 was positively correlated with sleep duration T1 and sleep duration T2 (*r* = 0.169 and 0.132, *p* < 0.001). Daily breakfast consumption T2 was positively correlated with sleep duration T1 and sleep duration T2 (*r* = 0.120 and 0.186, *p* < 0.001) (Table 3).

### 3.4. Cross-Lagged Model of Daily Breakfast Consumption, Sleep Duration and Depressive Symptoms

**Figure 1:**
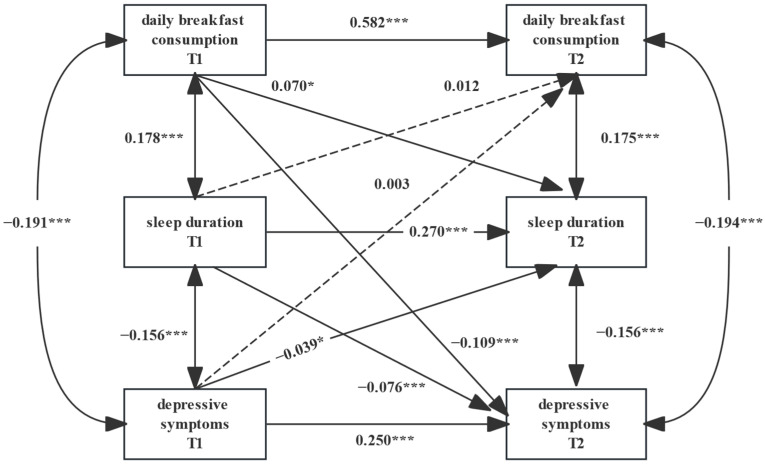
Cross-lagged model of daily breakfast consumption, sleep duration and depressive symptoms at baseline (T1) and follow-up (T2) with standardized path coefficients. Note: * *p* < 0.05, *** *p* < 0.001; T1 = assessed at baseline, T2 = assessed at follow-up. Solid lines indicate significant effects, while dashed lines indicate non-significant effects.

Figure 1 shows the results of the cross-lagged model for daily breakfast consumption, sleep duration and depressive symptoms. After controlling for sex, school type, academic performance, monthly pocket money, parental educational level, BMI, screen time and physical activity, the results of autoregressive path analysis for the same variable at different time points indicated that the autoregressive coefficients for daily breakfast consumption, sleep duration and depressive symptoms were 0.582, 0.270, and 0.250, respectively (all *p* < 0.001). Results from the cross-lagged path analysis indicated that daily breakfast consumption T1 positively predicted sleep duration T2 (*β* = 0.070, SE = 0.028, *p* =0.013) and negatively predicted depressive symptoms T2 (*β* = −0.109, SE = 0.030, *p* < 0.001). Sleep duration T1 negatively predicted depressive symptoms T2 (*β* = −0.076, SE = 0.022, *p* < 0.001), while depressive symptoms at T1 negatively predicted sleep duration T2 (*β* = −0.039, SE = 0.018, *p* = 0.028).

## 4. Discussion

The present study analyzed the dynamic changes in daily breakfast consumption, sleep duration and depressive symptoms in adolescents, explored the longitudinal associations between daily breakfast consumption, sleep duration and depressive symptoms. Results showed that the rate of daily breakfast consumption decreased, alongside the prevalence of depressive symptoms and insufficient sleep increased. Furthermore, daily breakfast consumption could negatively predict depressive symptoms and positively predict sleep duration. Sleep duration and depressive symptoms might have a bidirectional association.

In the current study, the rate of daily breakfast consumption among adolescents at T1 was 81.10%, which decreased to 76.06% at T2. The proportions were higher than the 71.0% found in Eastern China [17], yet lower than the 85.6% reported in a nationwide survey of Chinese adolescents aged 12–17 years from 2015 to 2017 [11]. Furthermore, this proportion was better than that reported in the United States (27.4%) [45] and some European countries (38.1–72.1%) [46]. Skipping breakfast might pose numerous health risks to individuals, such as an increased risk of obesity, hypertension, cardiovascular disease, diabetes and other chronic diseases [17]. Stratified analysis showed that the daily breakfast consumption rate among junior high school students showed a declining trend, while no significant difference was observed among senior high school students. This may be attributed to variations in eating self-regulatory capacity. Previous research indicated that adolescents experience a more pronounced decline in eating self-regulation from early adolescence (10–12-year-old) to mid-adolescence (14–16-year-old), with a slow recovery observed in late adolescence (17-year-old) [47]. And eating behaviors in mid-adolescence become more aligned with those of adulthood [48]. Therefore, it is essential to initiate monitoring and intervention of breakfast behaviors at the earliest possible stage, as this plays a critical preventive role in fostering healthy eating habits during later adolescence and adulthood.

This study revealed that 89.53% of the students in T1 and 92.89% of the students in T2 failed to meet the sleep duration recommended by the Ministry of Education of China. This finding is consistent with the data presented in a 2019–2020 Chinese national survey, which shows that 90.8% of junior high school students sleep for less than 9 h and 84.1% of senior high school students sleep for less than 8 h [16]. After the school type grouping, the follow-up changes in the insufficient sleep rate among senior high school students were not significant. The sleep duration of teenagers may be affected by factors such as puberty, electronic media use, school start time, and family factors, and can lead to many adverse effects, such as poor judgment, inattention, emotional imbalance, reduced decision-making ability, and obesity [49]. Sleep insufficiency is a prevalent phenomenon among Chinese adolescents. It is necessary to focus on the sleep problems of adolescents and provide guidance and intervention as early as possible.

The prevalence of depressive symptoms in this study was 15.31% at T1 and 18.47% at T2, indicating an increase. Both rates were slightly higher than the 14.8% in the Report on the Development of National Mental Health in China (2021–2022) [50]. In the current study, the prevalence of depressive symptoms was higher in girls than in boys, which is consistent with previous research findings [3,4]. Notably, the prevalence of depressive symptoms among boys increased significantly at follow-up. This might be because boys typically experience puberty later than girls. Furthermore, there are gender differences in stress-coping strategies. Boys are more likely to employ avoidance strategies, whereas girls tend to seek social support to address challenges [51]. Meanwhile, the prevalence of depressive symptoms among junior high school students increased from 13.74% to 17.31%, while no statistically significant change was observed among senior high school students in this study. This may be attributed to junior high school students potentially having weaker emotional regulation abilities compared to senior high school students. Research has shown that emotional regulation strategy scores among children and adolescents aged 9–12 were slightly lower than those among adolescents aged 13–16 [52]. Depressive symptoms in childhood and adolescence often persist into adulthood, imposing lifelong burdens on individuals [53]. Therefore, greater emphasis should be placed on adolescent mental health, establishing effective screening mechanisms and preventive measures at individual, school, and societal levels.

This study indicated that daily breakfast consumption negatively predicted subsequent depressive symptoms. Breakfast is considered to be an important influencing factor of depressive symptoms [54]. Previous research showed that children and adolescents who skip breakfast have about a threefold increased risk of depression [53], and skipping breakfast may contribute to the persistence of depression [55]. Several studies have also found a longitudinal effect of breakfast on depressive symptoms. Gong et al. found that skipping breakfast is potentially associated with later emotional symptoms [21]. Xu et al. also discovered a longitudinal association between breakfast and depressive symptoms among middle school students [23]. Another prospective study conducted among college students also revealed that the less frequently students ate breakfast, the higher the risk was of developing depressive symptoms the following year [56]. However, inconsistent with our findings, a previous study has failed to find a longitudinal association between breakfast consumption and depressive symptoms [22]. These inconsistencies may stem from differences in follow-up intervals or survey methodologies. Thus, future research with varied time interval, investigation methods, and population samples is warranted to clarify this relationship. Moreover, no predictive effect of depressive symptoms on daily breakfast consumption was found in this study. Nevertheless, previous research in adults has shown that depressive symptoms may have a predictive effect on health-related behaviors (including unhealthy eating behaviors) [57]. Therefore, further studies should extend the scope to examine the longitudinal associations between multiple unhealthy behaviors and depressive symptoms among adolescents.

The results of this study showed that sleep duration at T1 could negatively predict depressive symptoms one and a half years later, and vice versa. Zheng et al. discovered through genetic evidence that short sleep duration (<6 h) significantly increases the risk of depression, and conversely, depression also significantly raises the occurrence risk of short sleep duration [58]. Previous studies have observed the bidirectional relationship between sleep problems (such as sleep disorders and sleep quality) and psychological problems. Marino et al. indicated that a significant bidirectional association existed between depressive symptoms and sleep disorders in both childhood and early adolescence [29]. Vazsonyi et al. found that the relationship between sleep quality and depressive and anxiety symptoms was bidirectional [30]. Given the limited evidence on the bidirectional relationship between sleep duration and depressive symptoms in adolescents, this study provides new empirical evidence. In addition, the results of this study suggested that daily breakfast consumption at T1 can positively predict sleep duration at T2. This further confirms the positive effect of breakfast consumption on sleep duration. Moreover, a previous study found that skipping breakfast and the duration of sleep may also have a combined effect on depressive symptoms. Students with both insufficient sleep and skipping breakfast had the highest risk of depression [4]. Given the multifaceted associations among breakfast consumption, sleep duration, and depressive symptoms, it is necessary to concurrently monitor adolescents’ sleep status, breakfast behaviors, and emotional well-being.

The strength of this study is that it used a longitudinal design. This study explored the changes in breakfast consumption, insufficient sleep and depressive symptoms among adolescents of different sexes and school types. A cross-lagged model was employed to preliminarily examine the longitudinal association of breakfast consumption, sleep duration and depressive symptoms among adolescents, providing new empirical evidence on the complex interplay between mental health and health behavior in adolescents.

This study also has several limitations. First, the use of self-reported data may be subject to recall bias. Second, only one follow-up assessment was conducted. Future studies should include multiple waves of data collection. Third, this study only focused on the frequency of breakfast consumption among adolescents, without examining other aspects, such as breakfast contents, quality, timing, or setting, etc. And the sleep factors only focused on duration. Fourth, confounding factors were not measured in this study, such as other lifestyle factors (e.g., dietary behaviors beyond breakfast) and physical health conditions. Fifth, this study was conducted across different seasons, and seasonal effects were not controlled. Future studies should account for seasonality by including multiple seasonal assessments. Finally, the participants in the study were adolescents in Shanghai, which cannot represent China as a whole. Future research could expand the sample selection scope to enhance sample representativeness.

## 5. Conclusions

This longitudinal study found that adolescents’ breakfast consumption, insufficient sleep and depressive symptoms exhibited unfavorable trends. The follow-up results indicated a decreasing proportion in daily breakfast consumption, alongside an increase in the prevalence of depressive symptoms and insufficient sleep. Furthermore, this study indicated that daily breakfast consumption could predict depressive symptoms and sleep duration among adolescents. Sleep duration and depressive symptoms might have a bidirectional relationship. This provides a new perspective for exploring the complex associations among these three factors. Based on these findings, it is recommended to closely monitor adolescents’ breakfast, emotional states, and sleep status. Establishing effective home-school collaboration mechanisms and enhancing nutrition and health literacy education for adolescents will enable early intervention programs, so as to foster a positive impact on physical and mental health among adolescents.

## Figures and Tables

**Table 1:** Demographic characteristics at baseline (T1)/*n* (%).

Variables	Total	Girls	Boys
**School type**			
Junior high school	1768 (70.66)	860 (68.25)	908 (73.11)
Senior high school	734 (29.34)	400 (31.75)	334 (26.89)
**Academic achievement**			
Top quarter	897 (35.85)	429 (34.05)	468 (37.68)
Middle	1057 (42.25)	565 (44.84)	492 (39.61)
Bottom quarter	548 (21.90)	266 (21.11)	282 (22.71)
**Monthly pocket money**			
Below ¥200	1715 (68.55)	841 (66.75)	874 (70.37)
¥200–599	556 (22.22)	321 (25.48)	235 (18.92)
¥600 or above	231 (9.23)	98 (7.78)	133 (10.71)
**Paternal education**			
Junior high school or below	412 (16.47)	191 (15.16)	221 (17.79)
Senior high school or equivalent	727 (29.06)	380 (30.16)	347 (27.94)
Junior college or equivalent	588 (23.50)	318 (25.24)	270 (21.74)
Bachelor’s degree or above	775 (30.98)	371 (29.44)	404 (32.53)
**Maternal education**			
Junior high school or below	496 (19.82)	236 (18.73)	260 (20.93)
Senior high school or equivalent	710 (28.38)	375 (29.76)	335 (26.97)
Junior college or equivalent	603 (24.10)	306 (24.29)	297 (23.91)
Bachelor’s degree or above	693 (27.70)	343 (27.22)	350 (28.18)
**BMI**			
Underweight	327 (13.07)	140 (11.11)	187 (15.06)
Normal	1633 (65.27)	971 (77.06)	662 (53.30)
Overweight or obese	542 (21.66)	149 (11.83)	393 (31.64)
**Physical activity (per week)**			
<7 days	1756 (70.18)	940 (74.60)	816 (65.70)
7 days	746 (29.82)	320 (25.40)	426 (34.30)
**Screen time (per day)**			
<2 h	1591 (63.59)	823 (65.32)	768 (61.84)
≥2 h	911 (36.41)	437 (34.68)	474 (38.16)

**Table 2:** The changes in daily breakfast consumption, insufficient sleep and depressive symptoms/*n* (%).

	Daily Breakfast Consumption			Insufficient Sleep			Depressive Symptoms		
	T1	T2	*p*	T1	T2	*p*	T1	T2	*p*
**Total**	2029 (81.10)	1903 (76.06)	<0.001	2240 (89.53)	2324 (92.89)	<0.001	383 (15.31)	462 (18.47)	0.001
**Sex**									
Girls	991 (78.65)	936 (74.29)	0.001	1176 (93.33)	1207 (95.79)	0.005	216 (17.14)	250 (19.84)	0.056
Boys	1038 (83.57)	967 (77.86)	<0.001	1064 (85.67)	1117 (89.94)	<0.001	167 (13.45)	212 (17.07)	0.005
**School type**									
Junior high school	1475 (83.43)	1376 (77.83)	<0.001	1586 (89.71)	1665 (94.17)	<0.001	243 (13.74)	306 (17.31)	0.002
Senior high school	554 (75.48)	527 (71.80)	0.053	654 (89.10)	659 (89.78)	0.704	140 (19.07)	156 (21.25)	0.227

Note: T1 = assessed at baseline, T2 = assessed at follow-up.

**Table 3:** Correlations between depressive symptoms, daily breakfast consumption and sleep duration.

	1	2	3	4	5	6
1. Depressive symptoms T1	1					
2. Depressive symptoms T2	0.321 ***	1				
3. Daily breakfast consumption T1	−0.195 ***	−0.156 ***	1			
4. Daily breakfast consumption T2	−0.130 ***	−0.220 ***	0.365 ***	1		
5. Sleep duration T1	−0.264 ***	−0.190 ***	0.169 ***	0.120 ***	1	
6. Sleep duration T2	−0.202 ***	−0.256 ***	0.132 ***	0.186 ***	0.411 ***	1

Note: *** *p* < 0.001. T1 = assessed at baseline, T2 = assessed at follow-up.

## Data Availability

The data presented in this study are available on request from the corresponding author due to ethical restrictions regarding minors.

## Abbreviations

PHQ-2-C	Chinese version of the Patient Health Questionnaire-2
BMI	Body Mass Index
WLSMV	Weighted Least Squares Mean and Variance adjusted
SE	Standard Error
SD	Standard Deviation
